# Sensitivity of Pegfilgrastim Pharmacokinetic and Pharmacodynamic Parameters to Product Differences in Similarity Studies

**DOI:** 10.1208/s12248-019-0349-3

**Published:** 2019-07-08

**Authors:** Ari Brekkan, Luis Lopez-Lazaro, Elodie L. Plan, Joakim Nyberg, Suresh Kankanwadi, Mats O. Karlsson

**Affiliations:** 1 Pharmetheus, Uppsala, Sweden; 2 Pharmacometrics Research Group, Department of Pharmaceutical Biosciences, Uppsala University, Uppsala, Sweden; 3 Dr. Reddy’s Laboratories, Basel, Switzerland

**Keywords:** biosimilarity, exposure sensitivity, granulocyte colony-stimulating factor, pegfilgrastim, population pharmacokinetic-pharmacodynamic modelling

## Abstract

In this work, a previously developed pegfilgrastim (PG) population pharmacokinetic-pharmacodynamic (PKPD) model was used to evaluate potential factors of importance in the assessment of PG PK and PD similarity. Absolute neutrophil count (ANC) was the modelled PD variable. A two-way cross-over study was simulated where a reference PG and a potentially biosimilar test product were administered to healthy volunteers. Differences in delivered dose amounts or potency between the products were simulated. A different baseline absolute neutrophil count (ANC) was also considered. Additionally, the power to conclude PK or PD similarity based on areas under the PG concentration-time curve (AUC) and ANC-time curve (AUEC) were calculated. Delivered dose differences between the products led to a greater than dose proportional differences in AUC but not in AUEC, respectively. A 10% dose difference from a 6 mg dose resulted in 51% and 7% differences in AUC and AUEC, respectively. These differences were more pronounced with low baseline ANC. Potency differences up to 50% were not associated with large differences in either AUCs or AUECs. The power to conclude PK similarity was affected by the simulated dose difference; with a 4% dose difference from 6 mg the power was approximately 29% with 250 subjects. The power to conclude PD similarity was high for all delivered dose differences and sample sizes.

## Introduction

Chemotherapy-induced neutropenia (CIN) is a major risk factor for infection-related morbidity and mortality, and also a significant dose-limiting toxicity in cancer treatment. Patients developing severe (grade 3/4) or febrile neutropenia (FN) during chemotherapy frequently receive dose reductions and/or delays to their chemotherapy. This may impact the success of treatment, particularly when treatment intent is either curative or to prolong survival (1). In patients undergoing cytotoxic chemotherapy, both primary and secondary prophylaxis with granulocyte colony-stimulating factors (GCSFs) have been demonstrated to reduce the incidence of severe and FN, length of neutropenia, neutropenia-associated complications, antibiotic use and the duration of hospitalization (2). Pegfilgrastim (PG) is a sustained-duration pegylated form of filgrastim, a recombinant methionyl form of human GCSF that is widely used in CIN management (3,4). Several biosimilar filgrastim (Neupogen®, Amgen) products have been approved by the European Medicines Agency (EMA) and US Food and Drug Administration (FDA) since 2008; however, the first approvals of PG biosimilars are very recent (5,6). One biosimilar PG product was approved by the FDA in June 2018 and five biosimilar PG products, including the product approved in the US, were recommended for approval by the EMA between July and September 2018 (6–8). Biosimilars are analogous to generics for small molecular weight drugs developed after the exclusive marketing period has expired although the regulatory requirements are very different due to the greater complexity of biological entities. Cost-effectiveness analyses have shown that the market entry of biosimilar filgrastim was associated with a treatment cost reduction (9,10). The approval of biosimilar PG will potentially also be associated with lower treatment costs thereby improving patient access to PG.

A key biosimilar development issue is the difficulty in ensuring consistency in manufacturing, purification, formulation, packaging and storing of the drug (11). Small differences in any of these steps may lead to molecular differences in the final product administered to patients. Since biologics are manufactured in living organisms, slight differences are always present between batches due to post-translational modification in the cell line or minute differences in bioreactor conditions. For a product to be considered biosimilar, inconsequential molecular differences between the product and the reference product are acceptable (and expected), but qualitative and/or quantitative differences need to be justified and demonstrated to be of no clinical significance (12).

To confirm clinical similarity, phase I studies are often performed comparing the pharmacokinetics (PK) and, if a suitable biomarker is available, pharmacodynamics (PD) of the potential biosimilar with those of the reference product using bioequivalence criteria. Two products are usually considered to be bioequivalent if the 90% confidence interval (CI) of the geometric mean ratios of PK parameters, such as area under the curve (AUC) and maximum concentration (Cmax) fall completely between 80 and 125% (13,14). For PD parameters, the geometric mean ratios of the area under the effect curve (AUEC) and maximum effect (Emax) are typically used. Several studies have demonstrated that some biosimilar PG candidates are comparable to the reference product in healthy volunteers using bioequivalence criteria (15–17). However, assessing PK and PD similarity using non-compartmental analysis (NCA) derived metrics such as AUC and Cmax may not be appropriate for biologics with non-linear disposition as it is more sensitive to influences depending on trial design (18). Additionally, AUC and Cmax might need to be corrected by differences in the protein content of the candidate biosimilar and the reference product. Correction for protein content may be acceptable on a case-by-case basis if pre-specified and adequately justified (19). These corrections usually assume a linear relationship between dose and AUC and Cmax, something which is inappropriate for drugs with non-linear disposition and could bias NCA results. A more informative method for similarity assessment is population PKPD modelling which may offer some degree of mechanistic understanding for observed differences in PK and PD that analyses such as NCA cannot provide even if current regulations consider that population PK data will not provide an adequate assessment for PK similarity (14). Although population PKPD models have been used in assessments of similarity earlier, their use in demonstrating sensitivity to external factors is seldom reported (20–22). For instance, model simulations can explore how much a potential biosimilar may differ with regard to delivered dose amount or potency from the reference product while still falling within the predefined bioequivalence criteria. Additionally, these tools can contribute to the selection of an optimally informative dose or doses for evaluating similarity (14).

An additional complicating factor in the development of biosimilars is immunogenicity, the potential for patients/subjects to form an immune response against the treatment (23–26). In addition to showing PK and PD equivalence, the immunogenic potential of a biosimilar must be comparable to that of the reference product (23,24,27). If anti-drug antibodies (ADA) are formed more or less readily against the biosimilar, the PK and PD will quite likely be affected. ADA are frequently included as time-static covariates in population PKPD analyses, but when the number of ADA positive subjects is very low, this is not always possible. In such cases, ADA positive records or individuals may be excluded from the analysis, but the exclusion rule may influence the analysis.

A population PKPD model describing the time-course of PG and ANC was previously developed (28). The PK and PD variables were PG concentration and ANC, respectively. The data used for model building were obtained from a three-way PK and PD similarity trial in healthy volunteers comparing a potential biosimilar PG (BIOS_PG) developed by Dr. Reddy’s Laboratories, India, with two different batches of the reference product (Neulasta®, Amgen), sourced from the US and EU, respectively. All products were administered subcutaneously at a dose of 6 mg. The model was a bidirectional mechanism-based model where the administration of PG influenced the neutrophil kinetic system resulting in an increase in ANC which subsequently increased the elimination of PG. Both PG and ANC were modelled; however, modelling of ANC was facilitated by prior model-related information being obtained from the literature minimizing the number of estimated parameters. The sensitivity of PK and PD parameters to small changes in dose was briefly explored in the previous publication.

The aims of this work were to use the previously developed model to answer the following questions related to the assessment of PK/PD similarity in a feasible study: (i) How important is the nominal administered dose for the assessment of PG PK/PD similarity? (ii) How closely does the delivered dose amounts of a reference and a test product have to match in order to conclude PG PK/PD similarity? (iii) What is the influence of ANC baseline for the assessment of PG PK/PD similarity? (iv) How sensitive is a PK/PD similarity study to eventual differences in PG product potency?

## Material and Methods

### Pharmacokinetic-Pharmacodynamic Model

A previously developed mechanism-based bidirectional population PKPD model coupling PG concentrations with ANC was used (28). Differential equations for the model are presented in Appendix 1. PK was described using a one-compartment model with dual PG absorption processes and parallel ANC-dependent and non-specific PG elimination. The observed PD variable that was modelled was ANC time-course which PG influenced by affecting the kinetic parameters describing the proliferation, maturation and margination of neutrophils and precursors. The CD34+ cell count in peripheral blood was also measured but is not included in this analysis. The model did not include covariate effects, as in the original model and data only little (∼ 2%) of the variability in AUCs could be explained by the tested covariates. In the original model, occasions where an individual had a confirmed ADA measurement against either the PEG moiety or the filgrastim moiety of the drug molecule were ignored but confirmed ADA for which it was not possible to ascertain the part of the molecule they bind to were kept in the model. An additional exploration of the influence of this exclusion method is presented in Appendix 2.

### Data

Data from a study with a two-way cross-over design were simulated where a nominal dose of PG was administered to healthy volunteers and compared with a product which was either identical or had a delivered dose or potency difference. Simulations were performed using 40 replicates of the trial population (*n* = 174 evaluable individuals) matching the one for which the model was originally developed, thus the total number of simulated individuals was 6960 (28). This number was deemed to be sufficiently large for the purpose of this work without creating an unnecessarily high computational burden of simulation and data management. Each simulated individual had PK and PD samples at 0, 1, 4, 6, 8, 10, 12, 14, 16, 18, 20, 24, 28, 32, 36, 40, 48, 60, 72, 96, 120, 168, 216, 264 and 312 h after the administered doses in the simulated dataset for a total of 50 observations per individual and administration.

### Simulations

Several scenarios were simulated to produce AUCs and AUECs from 0 to 312 h after the given doses in all individuals to gauge model sensitivity. AUCs and AUECs were based on the PG concentration-time and ANC-time curves, respectively. In the first scenario, individuals receive a PG dose of 2, 3, 4 or 6 mg and a product with the same nominal dose with a 0, 2, 4, 6, 8 and 10% difference in delivered (bioavailable) dose amount representing the amount of drug reaching the systemic circulation. Since the model used for simulation only included a single 6 mg dose level, cautious extrapolations to the other lower doses are performed. The same simulation as above for the 6 mg dose level, with a different baseline ANC value, was also performed in the second scenario. In this evaluation, the sensitivity to dose changes in the presence of baseline ANC values of 2.7 and 0.5 billion cells/L were compared. A third scenario was simulated where individuals received a PG dose of 6 mg followed by a product with a 0, 5, 50, 500, 1000 and 5000% difference in potency, achieved by multiplication of the individual EC50 value. Complete washout between administrations was assumed. The geometric means of the AUCs and AUECs based on all 6960 individuals were calculated for each of the simulated scenarios.

### Statistical Power to Conclude PK and PD Similarity

The statistical power in the simulated study to conclude PK and PD similarity was calculated by comparing ratios of the geometrical means of the AUCs and AUECs between the two products given differences in delivered doses or potency between the products. While traditional PK similarity criteria for drugs not administered intravenously require the demonstration of equivalence in AUC and Cmax, AUC and AUEC were the only considered metrics in this work. A bioequivalence criteria of having the 90% CI of the AUC or AUEC ratio falling between 80 and 125% was used. The CI was calculated using the *t*-statistic with *n*-1 degrees of freedom where *n* is the number of individual AUCs or AUECs. The expected power of PK and PD similarity was calculated as the percentage of the simulated studies that were PK or PD equivalent according the set criteria. A total of 10,000 studies were simulated, each by bootstrap sampling of individuals from the simulated scenarios.

### Data Analysis and Software Details

NONMEM version 7.3.0 was used for data simulation (29). Perl-speaks-NONMEM (PsN) version 4.7.0 was used for NONMEM run control (30,31). Data management, generation of simulation models and plotting of results were performed using R version 3.3.3 (32).

## Results

### Simulations

**Fig. 1:**
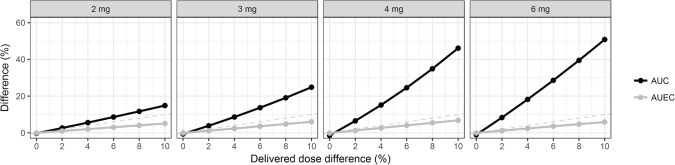
Geometric mean area under the pegfilgrastim concentration-time curve (AUC, black) and geometric mean area under the ANC-time curve (AUEC, grey) differences *versus* difference in delivered dose, stratified by nominal dose. The grey dashed line is an identity line

The differences in AUCs and AUECs resulting from delivered dose differences between the reference and test products are illustrated for the four nominal doses (2, 3, 4 and 6 mg) in Fig. 1. Since the model used for simulation only included a single 6 mg dose level, the simulations of lower doses can be considered to be explorative. There was a trend that delivered dose differences had a larger influence on AUCs for larger nominal doses while AUECs were relatively unaffected by small dose differences across the nominal doses. The AUC differences for a 10% difference were 15, 25, 46 and 51% for the 2, 3, 4 and 6 mg doses, respectively. The AUEC differences for a 10% delivered dose difference were all ≤ 7%, with the largest difference observed for the 4 mg dose (7%).

**Fig. 2:**
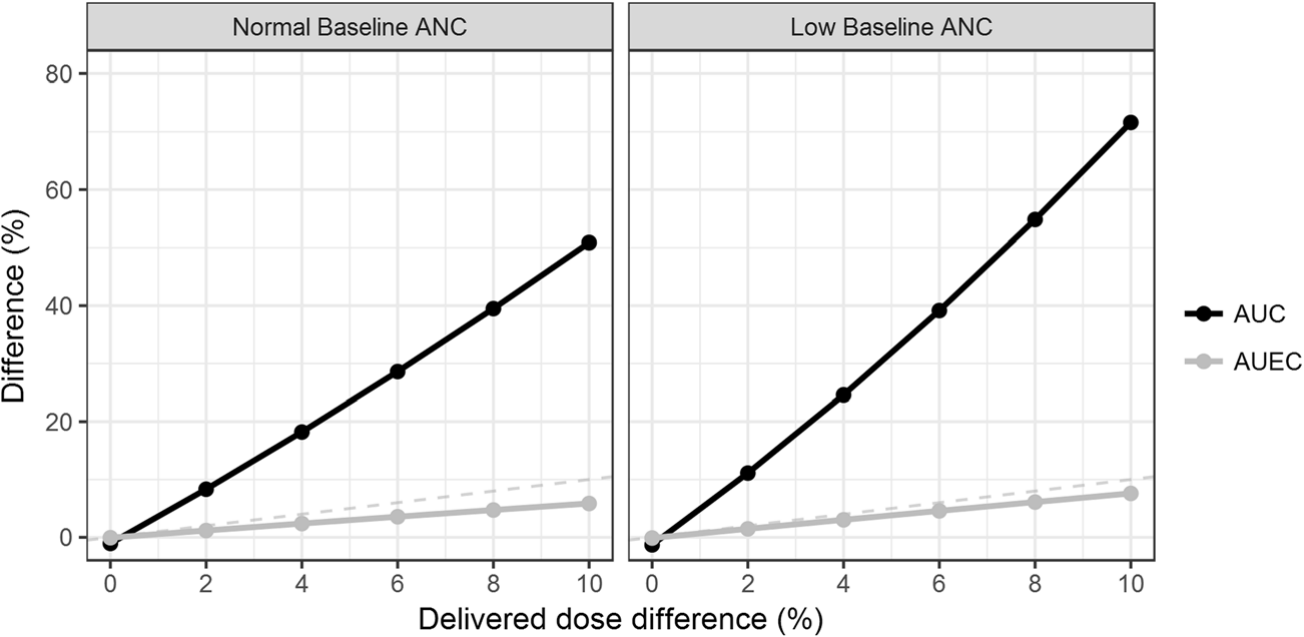
Geometric mean area under the pegfilgrastim concentration-time curve (AUC, black) and geometric mean area under the ANC-time curve (AUEC, grey) differences between a 6 mg reference and test product *versus* differences in delivered dose between the products. The plot has been stratified by different baseline absolute neutrophil counts (ANC). The grey dashed line is an identity line

The effect of having a lower baseline ANC following the administration of a nominal 6 mg dose on the PK and PD sensitivity is presented in Fig. 2. When baseline ANC was low (0.5 billion cells/L), the AUC difference was 72% for a 10% dose difference compared with an AUC difference of 51% for a normal ANC baseline (2.7 billion cells/L).

**Fig. 3:**
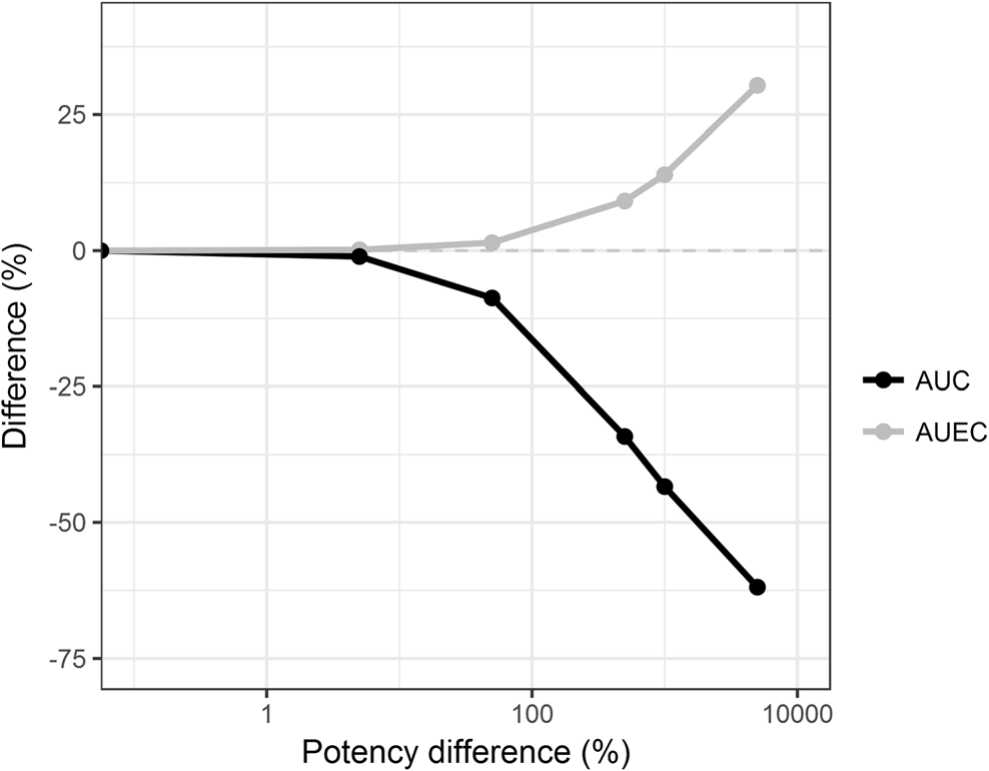
Geometric mean area under the pegfilgrastim concentration-time curve (AUC, black) and geometric mean area under the ANC-time curve (AUEC, grey) differences between a 6 mg reference and test product *versus* differences in potency. The grey horizontal dashed line indicates no difference in AUC or AUEC

The influence of different potencies between the reference and test products on AUCs and AUECs is presented in Fig. 3. The influence of potency differences between the products was most apparent on the AUECs. Potency differences of ≤ 50% resulted in differences of ≤ 2% and ≤ 15% in AUC and AUEC, respectively. The impact of potency differences on AUEC for potency differences larger than 50% resulted in a maximum difference of around 65%.

### Statistical Power to Conclude PK and PD Similarity

**Fig. 4:**
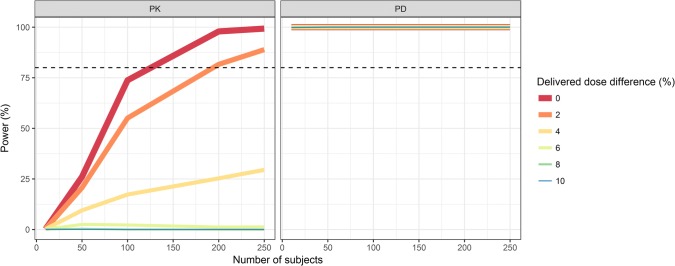
Statistical power to conclude pharmacokinetic (PK, left panel) and pharmacodynamic (PD, right panel) similarity in a study with a two-way cross-over design with delivered dose differences between the reference and test products ranging from 0 to 10%. The nominal dose administered was 6 mg. The horizontal dashed line indicates 80% power

The statistical power to conclude PK and PD similarity based on AUC and AUECs, respectively, in the 6 mg cross-over trial is presented in Fig. 4. The power to conclude PD similarity was almost 100% while the power to conclude PK similarity depended on delivered dose differences. The larger the delivered dose difference between the two products, the larger the sample size required to conclude PK similarity. With a 6 mg dose, ∼ 125 individuals resulted in 80% power to conclude PK similarity with identical products in the cross-over study. With a 2% delivered dose difference the number of subjects needed to reach a power of 80% increased to ∼ 200.

**Fig. 5:**
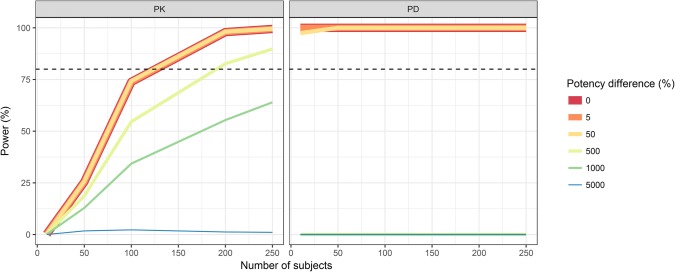
Statistical power to conclude pharmacokinetic (PK, left panel) and pharmacodynamic (PD, right panel) similarity in a study with a two-way cross-over design with potency differences between the reference and test products ranging from 0 to 5000%. The nominal dose administered was 6 mg. The horizontal dashed line indicates 80% power

The same power analysis performed with potency differences between the reference and test products is presented in Fig. 5. A potency difference ≥ 500% between the products resulted in a statistical power to conclude PD similarity that was null for all of the tested sample sizes. One hundred twenty-five individuals were necessary to reach 80% power when the potency was not more different than 50%. A 50% difference in potency was similar to the products being identical with regard to the power to conclude PK and PD similarity, but larger potency differences resulted in < 80% power to conclude PK similarity with the tested sample sizes.

## Discussion

The entrance of biosimilar filgrastim products in the European Economic Area reduced the daily treatment price by between 8 and 62% (33). The result of such price reductions may be improved patient access to the therapies and a reduction of the impact of CIN (33). The approval of biosimilar PG has been comparatively slow, with the first approval in either the US or the EU coming in June 2018 (6). Difficulties in developing biosimilar PG may be explored using methods outlined in this work.

*In silico* model simulation is a useful tool to assess scenarios which cannot be tested *in vitro* or *in vivo* provided that a well-performing model has been developed for the therapeutic in question. Model simulations have been used previously to determine the extent that products in a bioequivalence trial can differ while fulfilling the traditional bioequivalence criteria and may be a promising method to evaluate factors influencing PK and PD similarity for protein therapeutics (34). Simulations from a previously developed bidirectional population PKPD model describing the time-course of PG concentrations and ANC were used in this work to demonstrate model sensitivity to different drug and patient-specific parameter perturbations potentially influencing PK and PD similarity (28). Although other variables such as CD34+ are accepted as surrogates of GCSF efficacy, the developed model only considered ANC, which was therefore used as the PD variable in this work (35). Peripheral blood counts of CD34+ cells are not a relevant biomarker for pegfilgrastim effect in its currently approved indications. Model simulations were used in the calculation of statistical power to conclude PK and PD similarity given dose differences and potency differences between a reference and potential biosimilar PG product.

This work was partly motivated by a three-way crossover PG PK/PD similarity trial where BIOS_PG was compared with two batches of the reference product, Neulasta®, sourced from the US and EU, respectively. This trial was used in the development of the previously developed population PKPD model (28). The trial showed PD similarity between the products but failed to demonstrate PK similarity. The results were subsequently analysed, and it was found that the delivered doses of BIOS_PG may have been higher than those of the reference products, potentially causing the trial to fail. The current work was performed to test this hypothesis and to evaluate other factors of importance for the development of biosimilar PG. The motivating trial and results are presented separately in Appendix 3.

A phase I two-way crossover study was simulated where individuals received two PG products, a reference product and a test product. Amount and potency differences between the products were simulated by changing the delivered dose amount or EC50 value of one of the administered products. The resulting geometric mean AUCs and AUECs of the population, based on PG concentration-time and ANC-time curves, respectively, were calculated and compared. Although PK similarity criteria require both Cmax and AUC to be within the specified limits, only AUCs were considered in this work. The results of the performed similarity trial with regard to either AUC or Cmax did not differ significantly (Appendix 3). Thus, for the purpose of this work, to demonstrate exposure sensitivity to differences in delivered dose, potency and baseline ANC values, AUCs and AUECs calculated from 0 to 312 h were deemed to be sufficient metrics of exposure and effect, respectively.

There was a substantial difference (> 8%) in the geometric mean AUC with a delivered dose difference of 2% between two 6 mg products indicating high PK parameter sensitivity to dose differences. For doses lower than 6 mg, the impact was somewhat smaller suggesting that it would be easier to achieve PK similarity with lower doses. However, these results are considered exploratory as the model performance for lower doses has not been evaluated. Comparing the exposure reported by Waller *et al*. (17) to the exposure range predicted by the present model following a 2 mg dose indicates an underprediction of exposure by the developed model which underscores the need to consider exploratory the lower dose data generated by this model. The geometric mean AUECs were much less affected by differences in the delivered dose; a 10% difference maximally resulted in an AUEC difference of 7%. The 6 mg dose had a 1% smaller AUEC difference than the 4 mg dose, likely an artefact of the stochasticity of the model simulations. Relevant differences in the clinical outcome induced by dose variations in this range are therefore unlikely (35). The saturable relationship between concentrations of PG and ANC may be responsible for the small influence that dose differences have on AUECs. The bidirectional nature of the system also plays a role here since higher PG concentrations likely have a shorter effect on the neutrophil production due to the increased ANC-dependent clearance of PG at higher concentrations. Together, these results indicate that a very tight control of the delivered doses and/or protein content of vials is crucial to demonstrate PK similarity of PG products (once delivered doses are matched, PK evaluations would remain sensitive to bioavailability variations). Protein content differences of up to ~ 14% between the reference and test products have been reported for biological therapies, necessitating post hoc dose corrections for similarity studies (36). Clearly, for PG, extra steps may be needed to ensure consistently delivered doses and/or protein content between the two products that are being compared in a similarity study.

The sensitivity of the AUCs and AUECs given different baseline ANC levels demonstrates the potential importance of considering ANC in the assessment of PK and PD similarity of PG where the differences between the products are more pronounced at lower baseline ANC levels. However, the low ANC that was simulated assumed severe neutropenia (cutoff point for grade 4 neutropenia = 0.5 billion cells/L (37)) which is observed in patients. Differences due to different baseline ANC in a PG biosimilar study performed in healthy volunteers would be smaller than those simulated in this work but may potentially be of consequence (38). Further, the model used for these simulations was built on healthy volunteer data where the lowest observed ANC was ~ 0.9 billion cells/L. In the patient situations, where lower ANC values are actually present, extra components of the system not identified in the current model can become relevant.

Potency differences between the reference and test products were simulated to determine the sensitivity of PG AUC and AUEC to potency differences. A 50% difference in potency (tested here for illustrative purposes but unlikely to be present in an actual study) had little influence on either AUCs or AUECs, where AUCs differed marginally (< 1.5%) while AUEC differences were 15% (*i*.*e*. changed less than linearly but still appreciably). These results demonstrate that the evaluation of PD similarity based on ANC in healthy volunteers is poorly sensitive to detect differences in product potency between a candidate PG biosimilar and the reference product.

Model simulations can be used to determine the number of individuals required to power a trial under different trial conditions to answer so-called “what if” questions (39,40). In this work, we answer two “what if” questions regarding the control of type-II errors in the simulated scenarios: (i) What happens to the statistical power to conclude PK or PD similarity given dose differences between a reference and test PG products? (ii) What happens to the statistical power to conclude PK or PD similarity given difference in potency (represented by perturbations of the EC50 parameter) between a reference and test PG products?

The statistical power to conclude PK similarity in the hypothetical trial was influenced by dose differences where a 10% dose difference resulted in ~ 0% power for the tested samples size (up to 250). Interestingly, for a dose difference of 4% or more from the nominal dose of 6 mg, trials would quite likely require an exorbitant number of individuals to be sufficiently powered to conclude PK similarity. In the trial outlined in Appendix 3, the number of individuals included in the main PK and PD populations was between 114 and 118 and the post hoc protein content difference between the reference PG and test PG was estimated to be between 5 and 8%. With a 4% dose difference, the power to conclude PK similarity was < 30% with an evaluable sample size of 250 individuals indicating that even if the number of individuals in the motivating trial would have been doubled the trial would likely not be sufficiently powered to conclude similarity. With no dose difference between 6 mg nominal doses of the reference and test products, the sample size would need to be ~ 125 to reach 80% power to conclude PK similarity. Recent similarity studies for PG report study sizes of 185 (15) and 172 (16) for a 6 mg dose which should be sufficient according to this analysis given negligible differences in delivered dose content between the products. However, the results of this work suggest that with a mere 2% difference in delivered dose, the number of patients recruited for those trials would not be sufficient to reach 80% power to conclude PK similarity.

When potency differences between the reference and test products were explored, it was determined that a sample size of ~ 125 would result in a statistical power to conclude PK similarity of 80% up to a potency difference of 50%. These results suggest that the test and reference products may differ substantially with regard to potency and still be considered to have similar PK. With a sample size of 50, the power to conclude PD similarity with a 50% potency difference was ~ 100% for all tested doses. Larger potency differences (500, 1000 and 5000%) resulted in a power to conclude PD similarity which was ~ 0% for all tested sample sizes. In reality, the potency of the biosimilar will not be as different from the reference product as those scenarios tested in this work, but the results suggest that AUC and AUEC are relatively insensitive to changes in PG potency and that within the actually expected variation ranges, a PKPD study in healthy volunteers is a more sensitive experiment to detect changes in the amount of delivered (bioavailable) protein to the circulation (the product of protein content and relative bioavailability) than to detect changes in product potency. Additionally, concerns over potency differences should be addressed prior to performing clinical trials in *in vitro* assays.

While this power analysis appears to answer the “what if” questions, it cannot address the number of dropouts, which would also influence any results (41). Power analyses can only provide evaluable samples sizes, but an additional number of patients would be recruited to account for drop-out in a real study. Additionally, the results of this analysis are also dependent on the IOV parameters estimated in the original data which were associated with relatively large uncertainty (relative standard errors > 40%) suggesting that the results could be conservative or not depending on the true IOV magnitude (28). Unfortunately, there is no way of knowing the true IOV magnitude, and thus the work is limited by the previous parameter estimates. An inflation of IOV magnitude would give a more conservative result with regard to power which may be recommended if little is known about the reference product.

All simulations in this work were performed using a model that fits the data to which it was developed well. However, that data were single nominal dose level data and previous modelling work have suggested a dose-dependent relative bioavailability for pegfilgrastim which is not accounted for in present work (42). Although the relative differences in bioavailability of the Roskos *et al.* study were small for doses up to 100 μg/kg, the consequence of such an omission may be significant given the sensitivity of the system (42). Without additional doses to re-evaluate the developed model to quantify any dose dependencies not accounted for, the extrapolations to lower doses than 6 mg can be regarded as exploratory. However, 6 mg is the approved dose for treatment of FN and most biosimilarity studies compare approved doses of the products and in simulations with the 6 mg dose extrapolations are not performed. Further, perfect capture of the AUCs and AUECs was assumed while it can be expected that a number of observations would fall below the quantification limit.

## Conclusions

A simulation-based analysis demonstrated that PK similarity as assessed by AUC of PG was sensitive to bioavailable dose, baseline neutrophil count and, to a lesser degree, the EC50 of the administered products while AUEC was only sensitive to large differences in EC50. To demonstrate PK similarity, differences in dose content between the reference and test product need to be very small while other tested differences were somewhat less influential. The results of this work demonstrate that several factors need to be controlled and considered in the assessment of biosimilars for PG and that model-based simulations can be a valuable tool to assess factors of importance for biosimilar development.

## Figures and Tables

**Fig. 6:**
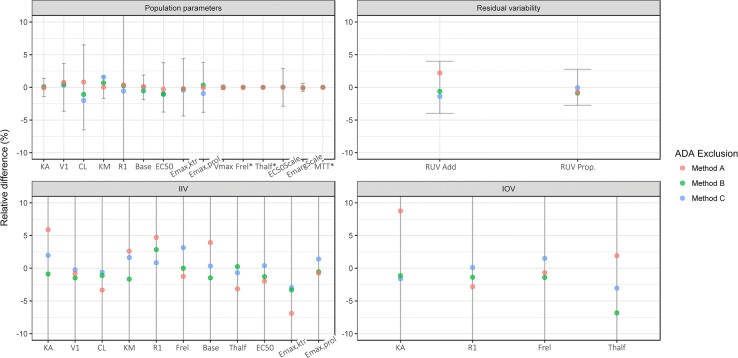
Relative difference from the original parameter estimates of population parameters, residual variability parameters, diagonal inter-individual variability (IIV) parameters and diagonal inter-occasion variability (IOV) parameters estimated using three different ADA exclusion rules. Error bars indicate relative standard errors of the original parameter estimates. *in the population parameters indicates fixed estimates

## Appendix 1

### Differential equations of model used for simulations

PK model:

$$ \frac{dA1}{dt}=-{K}_a\cdot A1+ Rate $$

$$ \frac{dA2}{dt}={K}_a\cdot F\cdot A1-\frac{Vmax/F\cdot {C}_{A2}}{KM+{C}_{A2}}-\frac{\left( CL/F\cdot \frac{C_{ANC}}{1000}\right)}{Vc/F}\cdot A2 $$

$$ {C}_{A2}=\frac{\left(A2\cdot 100000\right)}{Vc/F} $$

$$ {C}_{\mathrm{ANC}}=\frac{A8}{V_{app}} $$

$$ {V}_{app}=1+\frac{Emarg\cdot {C}_{A2}}{EC50 marg+{C}_{A2}} $$

where *A*1 is the amount of pegfilgrastim in the absorption compartment, and *A*2 is the amount of pegfilgrastim in the central compartment, *K*_a_ is the first-order absorption rate, *F* is bioavilability, *V*max/*F* is the maximum saturable elimination rate, *KM* is the Michaelis-menten constant, *V*c/*F* is the central volume, *C*_*A2*_ is the concentration of pegfilgrastim in the central compartment (in ng/L) and *C*_*ANC*_ is the ANC. The input to the absorption compartment is dictated by a zero-order input (*Rate*). *Emarg* and *EC50marg* are the maximum effect on margination and concentration of pegfilgrastim eliciting half the maximum effect on margination, respectively.

PD model:

$$ \frac{dA3}{dt}=A3\cdot {Prol}_{in}-{Prol}_{out}\cdot A3 $$

$$ \frac{dA4}{dt}=A3\cdot {Prol}_{out}-{K}_{tr}\cdot A4 $$

$$ \frac{dA5}{dt}=A4\cdot {K}_{tr}-{K}_{tr}\cdot A5 $$

$$ \frac{dA6}{dt}=A5\cdot {K}_{tr}-{K}_{tr}\cdot A6 $$

$$ \frac{dA7}{dt}=A6\cdot {K}_{tr}-{K}_{tr}\cdot A7 $$

$$ \frac{dA8}{dt}=A7\cdot {K}_{tr}-{K}_{out}\cdot A8 $$

$$ {E}_{trans}=\frac{E\max_{trans}\cdot {C}_{A2}}{EC50+{C}_{A2}} $$

$$ {E}_{prol}=\frac{Ema{x}_{prol}\cdot {C}_{A2}}{EC50+{C}_{A2}} $$

$$ {Prol}_{in}=\frac{5}{MTT}\cdot \left(1+{E}_{prol}\right) $$

$$ {Prol}_{out}=\frac{5}{MTT}\cdot \left(1+{E}_{prol}\right) $$

$$ {K}_{tr}=\frac{5}{MTT}\cdot \left(1+{E}_{tr ans}\right) $$

$$ {K}_{out}=\frac{LN(2)}{\mathrm{Neutrophil}\ {t}^{\frac{1}{2}}} $$

where *A*3 to *A*7 are the amounts (ANC) in the transit chain, *Prol*_*in*_ and *Prol*_*out*_ dictate input and output from the proliferation compartment (*A3*), respectively (in the simulations they were set to be equal). *K*_tr_ is the effective transit rate (with no drug effect it is equal to 5/*MTT*, the mean transit time), *E*_*trans*_ is the effect of pegfilgrastim on the transit rate (modelled with an Emax model), *E*_*prol*_ is the effect of pegfilgrastim on the proliferation rate (modelled with an Emax model), *K*_*out*_ is the neutrophil elimination rate. *A*8 is the amount in the ANC compartment.

Model structure and parameter estimates used for simulation are presented in (28).

## Appendix 2

In the original model, occasions where an individual had a confirmed ADA measurement against either the PEG moiety or the filgrastim moiety of the drug molecule were ignored but confirmed ADA for which it was not possible to ascertain the part of the molecule they bind to were not considered in the exclusion. The influence of the ADA exclusion method on model performance was evaluated testing three alternative rules of data exclusion. Rule A included no ADA exclusions, rule B excluded individuals that had a confirmed ADA results against the PEG or the filgrastim moieties on the drug molecule entirely from the analysis and rule C excluded any individuals with a confirmed ADA measurement, regardless of whether it was known which part of the PG molecule the ADA was formed against. The impact on model performance was evaluated by calculating the relative change from the original model parameter estimates.

Results of the ADA-based exclusion rule evaluation are presented in Appendix Fig. 6. The number of observation records for the different rules of ADA exclusion were 16,343, 16,635, 15,981 and 15,237 for the original rule of ADA exclusion and rules A, B and C, respectively. The maximum absolute relative difference in the population parameters from the original rule of ADA exclusion was 0.8, 0.7 and 1.6% for rules A, B and C, respectively. The largest parameter differences were observed for diagonal interindividual and interoccasion variability (IIV and IOV) parameters and exclusion rule A where the maximum absolute relative differences from the original rule of ADA-based exclusion were 5.8 and 8.8% for IIV and IOV parameters, respectively.

In the previously developed model, occasions where ADA were confirmed and qualified were excluded which may have influenced model parameters. ADA may play a role in the assessment of PK and PD similarity of protein therapeutics and differences in immunogenicity between the reference and test products need to be considered (24–26). Different ADA exclusion methods tested in this work illustrate an inconsequential impact on model parameter estimates. Although parameters estimated with the developed model appear to be robust to different ADA exclusion methods, the results presented herein may only be applicable to the drug and dose tested. For protein therapeutics which are more immunogenic, exclusion of ADA positive individuals may not be possible, and model-based methods for handling them would need to be employed. Moreover, as it usually takes some time or multiple doses for ADA to appear, a relevant part of the PK and PD profile after the first dose is likely to reflect the PK and PD of the drug in the absence of ADA, which may conversely affect the full profile after further doses. Probably due to these reasons, current EMA and FDA guidelines recommend the use of parallel arm rather than crossover designs for the evaluation of substances with a high risk of immunogenicity (14,19). In some development programs based in crossover studies, complementary immunogenicity studies with a parallel arm design might be needed to fully assess this issue.

## Appendix 3

**Table I:** Area under the pegfilgrastim concentration-time curve (AUC) and area under the ANC-time curve (AUEC) results of the motivating trial which failed to conclude PK similarity between BIOS_PG and the comparator batches (sourced from either the US or EU)

Treatment^a^	*n*	Geometric mean AUC^b^ [0-inf] (ng*h/mL)		Comparisons		
			95% CI^c^	Pair	Ratio (%)	90% CI^c^
BIOS_PG (A)	115	5093	(4327, 5994)	A/B	121.99	(109.09, 136.41)
US_PG (B)	114	4175	(3545, 4916)	A/C	130.67	(116.91, 146.04)
EU_PG (C)	116	3898	(3312, 4586)	B/C	107.12	(95.82, 119.75)
		Geometric mean AUEC^b^ [0-inf] (10^4^ *h/mm3)				
BIOS_PG (A)	115	7556	(7278, 7844)	A/B	103.34	(101.47, 105.24)
US_PG (B)	116	7312	(7043, 7590)	A/C	103.01	(101.15, 104.89)
EU_PG (C)	118	7335	(7066, 7614)	B/C	99.68	(97.89, 101.50)

^a^*BIOS_PG* potential biosimilar pegfilgrastim (PG), *US_PG* US-sourced reference PG, *EU_PG* EU-sourced reference PG

^b^*AUC = AUC* area under the PK (PG concentration-time) curve, *AUEC* area under the PD (ANC-time) curve

^c^*CI* Confidence interval

**Table II:** Maximum pegfilgrastim concentration (Cmax) and ANC (Emax) results of the motivating trial which failed to conclude PK similarity between BIOS_PG and the comparator batches (sourced from either the US or EU)

Treatment^a^	*n*	Cmax^b^ (ng/mL)		Comparisons		
			95% CI^c^	Pair	Ratio (%)	90% CI^c^
BIOS_PG (A)	121	146.9	(126.3, 170.8)	A/B	120.15	(107.83, 133.87)
US_PG (B)	121	122.2	(105.1, 142.1)	A/C	130.78	(117.37, 145.73)
EU_PG (C)	121	112.3	(96.57, 130.6)	B/C	108.85	(97.68, 121.30)
		Emax^b^ (10^4^/mm3)				
BIOS_PG (A)	114	38.49	(36.88, 40.17)	A/B	104.75	(101.82, 107.76)
US_PG (B)	118	36.74	(35.22, 38.34)	A/C	105.02	(102.07, 108.05)
EU_PG (C)	118	36.65	(35.13, 38.24)	B/C	100.26	(97.48, 103.11)

^a^*BIOS_PG* potential biosimilar pegfilgrastim (PG), *US_PG* US-sourced reference PG, *EU_PG* EU-sourced reference PG

^b^*Cmax* maximum PG concentration, *Emax* maximum absolute neutrophil count (ANC)

^c^*CI* Confidence interval

**Table III:** Protein content analysis results from the motivating trial

Product^a^	Mean protein content in mg (sd^b^)	Median protein content	Number of samples
US_PG	6.3 (0.10)	6.3	10
EU_PG	6.2 (0.09)	6.2	10
BIOS_PG (Batch 1)	6.7 (0.15)	6.7	10
BIOS_PG (Batch 2)	6.5 (0.33)	6.7	10
BIOS_PG (Combined batches)	6.6 (0.27)	6.7	20

^a^*BIOS_PG* potential biosimilar pegfilgrastim (PG), *US_PG* US-sourced reference PG, *EU_PG* EU-sourced reference PG

^b^*SD* sample standard deviation

An NCA analysis performed on the results of a PK and PD similarity trial for BIOS_PG concluded that BIOS_PG showed PD similarity to the reference products (US-sourced and EU-sourced Neulasta®) but failed to meet the criteria for PK similarity. In Appendix Tables I and II, the results for pegfilgrastim and ANC AUC and Cmax, respectively, corresponding to the main analysis population are presented. In general, the results based on AUC and Cmax were comparable. As shown in Appendix Tables I and II, the comparisons of Cmax (for PK)/Emax (for ANC) and AUC (for PK) / AUEC (for PD) between the products gave overall similar results. Further details of the conducted study are presented in (28).

Post hoc analyses to determine the protein content in the vials administered to subjects in the study found that the median protein content in BIOS_PG vials may have been between 6 and 8% higher than the reference batches (Appendix Table III). The protein content was obtained by 10 random samples from vials of the reference product and two separate BIOS_PG batches, respectively.
